# Human Adipose-Derived Stem Cells’ Paracrine Factors in Conditioned Medium Can Enhance Porcine Oocyte Maturation and Subsequent Embryo Development

**DOI:** 10.3390/ijms22020579

**Published:** 2021-01-08

**Authors:** Seok Hee Lee

**Affiliations:** 1 Department of Theriogenology and Biotechnology, College of Veterinary Medicine, Seoul National University, Seoul 08826, Korea; seokhee.lee@ucsf.edu; Tel.: +1-4154760932; 2 Center for Reproductive Sciences, Department of Obstetrics and Gynecology, University of California San Francisco, San Francisco, CA 94143, USA

**Keywords:** conditioned medium, enzyme-linked immunosorbent assay, growth factors, human adipose-derived stem cells, oocyte development

## Abstract

An essential requirement for the success of in vitro maturation (IVM) of the oocyte is to provide an optimal microenvironment similar to in vivo conditions. Recently, somatic cell-based coculture or supplementation of a conditioned medium during IVM has been performed to obtain better quality of oocytes, because they mimic the in vivo reproductive tract by secreting paracrine factors. In this study, human adipose-derived stem cells (ASC) and their conditioned medium (ASC-CM) were applied to IVM of porcine oocytes to evaluate the effectiveness of ASC on oocyte development and subsequent embryo development. In results, both ASC and ASC-CM positively influence on oocyte maturation and embryo development by regulating growth factor receptors (*VEGF*, *FGFR*, and *IGFR*), apoptosis (*BCL2*), cumulus expansion (*PTGS2*, *HAS2*, and *TNFAIP6*), and oocyte maturation-related genes (*GDF9* and *BMP15*). In particular, the fluorescence intensity of GDF9 and BMP15 was markedly upregulated in the oocytes from the ASC-CM group. Furthermore, significantly high levels of growth factors/cytokine including VEGF, bFGF, IGF-1, IL-10, and EGF were observed in ASC-CM. Additionally, the ASC-CM showed active scavenging activity by reducing the ROS production in a culture medium. Consequently, for the first time, this study demonstrated the effect of human ASC-CM on porcine oocyte development and the alteration of mRNA transcript levels in cumulus–oocyte complexes.

## 1. Introduction

Oocyte in vitro maturation (IVM) is a promising assisted reproduction technique (ARTs) for studying embryo development, animal model research, and the treatment of infertility [1,2,3]. An essential requirement for the success of IVM of the mammalian oocyte is to provide a rich microenvironment for oocyte culture, mimicking the in vivo reproductive tract [4,5]. Despite great efforts to the establishment of optimal culture medium conditions [6,7], numerous studies still have been performed to seek new additives [8,9,10] or somatic cell-based coculture systems [4,11] to obtain a better quality of oocytes. The somatic cell coculture system removes reactive oxygen species (ROS) and provides paracrine factors, which gives beneficial effects on oocyte development [12]. Previous studies have shown that the application of a cell-based coculture system can mimic the in vivo microenvironment by secreting various kinds of growth factors [13,14,15]. However, since there are numerous kinds of factors such as cytokines and growth factors, applying all these factors into an IVM system is rather high-priced. Moreover, the application of the coculture system during IVM would become cumbersome and laborious works due to maintaining the ideal condition of coculture cells before IVM. Additionally, the coculture cells still have the possibility to be contaminated until applying to IVM.

Recent studies suggested that stem cell-derived factors, called “biomaterials”, could improve the development of preimplantation embryos [16,17]. The secreted factors derived from the stem cells in the medium are referred to as extracellular vesicles [18]. Therefore, the medium is named the conditioned medium (CM) [19]. Previous studies have demonstrated that stem cells are able to secrete multiple factors such as the hepatocyte growth factor, granulocyte and macrophage colony stimulating factors, interleukins (IL), tumor necrosis factor-alpha, and vascular endothelial growth factor (VEGF) [20,21,22]. In addition, the various secreted factors derived from stem cells have been shown to be applied as reliable therapies for the treatment of wounds [23,24,25]. Therefore, it can be speculated that the supernatant of stem cells, which contains a large amount of growth factors, could provide a beneficial effect on oocyte development. With regard to stem cell-enhanced oocyte culture systems, several studies have shown a beneficial effect upon oocyte development. For instance, Jafarzadeh et al. [26] demonstrated that supplementation of IVM medium with 50% human bone marrow mesenchymal stromal cells-conditioned media significantly improved cytoplasmic and nuclear maturation of the germinal vesicle stage of murine oocytes. Additionally, they proved the conditioned media have a promising ability to improve IVM of polycystic ovary syndrome oocytes. In addition, human umbilical cord stem cells derived conditioned medium showed potential efficacy in terms of improving oocyte maturation and mRNA expression related to apoptosis [27].

**Figure 1:**
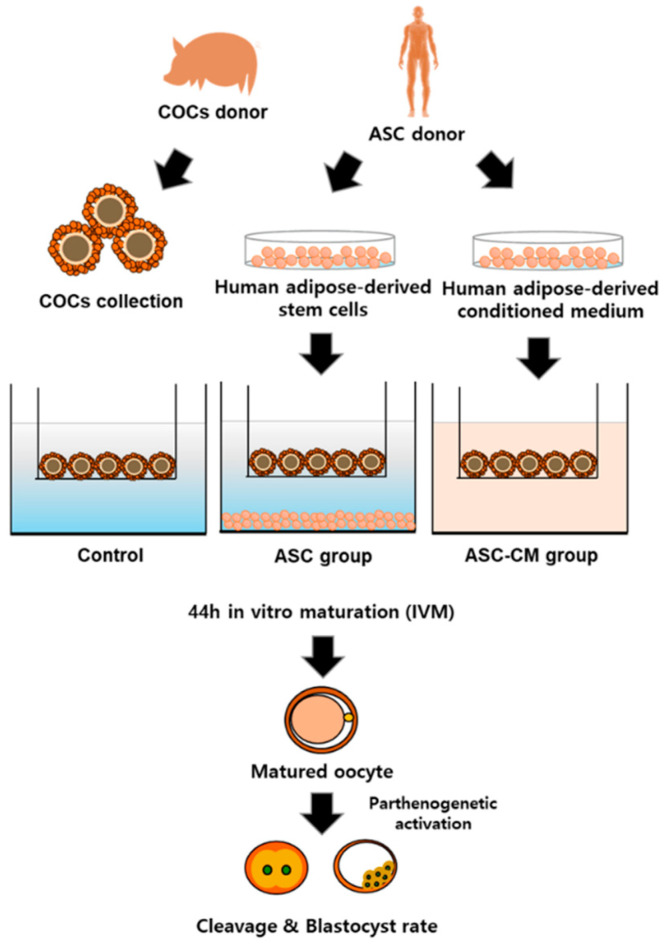
A schematic illustration of the experiments. Adipose-derived stem cell (ASC) coculture or supplementation of ASC-conditioned medium (CM) was applied to the porcine in vitro maturation (IVM) system. After 44 h of in vitro maturation, 1st polar body extrusion from oocytes and subsequent cleavage and blastocyst rate were assessed. ASC; human adipose-derived stem cells, ASC-CM; human adipose-derived stem cells conditioned medium.

Notably, adipose-derived stem cells (ASCs), a kind of mesenchymal stem cells, have been considered as an ideal candidate in regenerative medicine because of their stable differentiation capacity, easy expandability, and simple isolation techniques to obtain appropriate amounts [28]. ASC secrete a variety of cytokines and growth factors, such as VEGF, IL-6, tumor necrosis factor (TNF)-β1, TNF-α, and so on [29,30]. In particular, as the human ASC and their secretory factors have been well-established [31,32,33] in the field of medicine, it will be more reliable to apply the human ASC and their factors on ARTs. Through these results, it can be hypothesized that the indirect approach of CM derived from human ASC might show a positive effect on oocyte development by paracrine effects. In addition, it is necessary to evaluate the competence of human ASC on the oocytes derived from other mammals prior to applying them in human ARTs in the future study. However, up to date, still little information is available on the biological effect of ASC-CM during IVM whether ASC-secreted growth factors/cytokine play a key role in cumulus–oocyte complexes (COCs) development. Therefore, the aim of the study is to use human ASC-conditioned media, as a supplement, to improve the IVM culture medium for oocyte development and subsequent embryo development in vitro in the view of the potential functions of secreted factors from ASC. A schematic illustration of the experiment in the present study was described in Figure 1.

## 2. Results

### 2.1. Effect of ASC and ASC-CM on Porcine Oocyte Maturation and Cumulus Expansion

**Figure 2:**
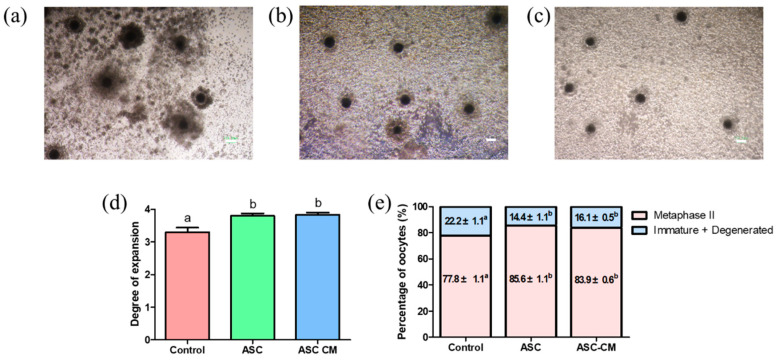
Effect of ASC/ASC-CM on oocyte development during IVM. (**a**) Expansion in cumulus cells from the control group. (**b**) Expansion in cumulus cells from the ASC group. (**c**) Expansion in cumulus cells from the ASC-CM group. (**d**) Degree of expansion in cumulus cells derived from three groups. (**e**) Percentage of matured oocytes or immature/degenerated oocytes derived from three groups. A total of 540 oocytes were used in the experiments. ^a,b^ Within a column, values with different superscripts are significantly different (*p* < 0.05). ASC; adipose-derived stem cell coculture group, ASC-CM; adipose-derived stem cell-conditioned medium group. The bar represents 100 μm.

To evaluate the effects of ASC coculture and ASC-CM on cumulus expansion of COCs and nuclear maturation of oocytes, the 1st polar body extrusion of oocytes and cumulus expansion degrees were assessed after 44 h of in vitro maturation. The proportion of COCs exhibiting complete cumulus expansion was significantly increased in ASC coculture and ASC-CM groups compared with the control (Figure 2a–d). As shown in Figure 2e, the nuclear maturation rate in ASC and ASC-CM groups (85.6% ± 1.1% and 83.9% ± 0.6%, respectively) was significantly higher than the control (77.8% ± 1.1%).

### 2.2. Quantification of Growth/Cytokine Factors from Culture Media

**Figure 3:**
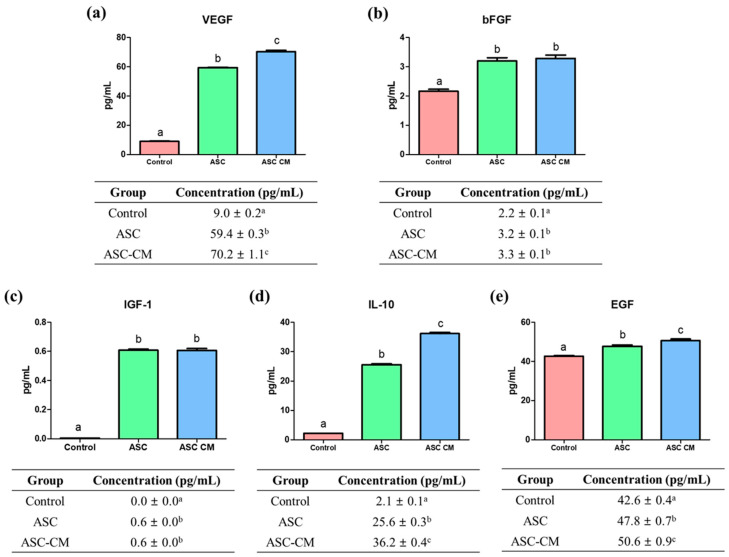
Concentration of (**a**) VEGF, (**b**) bFGF, (**c**) IGF-1, (**d**) IL-10, and (**e**) EGF from supernatant in each group. ^a,b,c^ Within groups, values with different superscript letters are significantly different (*p* < 0.05). Data are shown as means ± SEM. At least six replications were performed. VEGF; vascular endothelial growth factor, bFGF; basic fibroblast growth factor, IGF-1; insulin growth factor-1, IL-10; interleukin 10, EGF; epidermal growth factor.

To evaluate the total concentrations of VEGF, bFGF, IGF-1, IL-10, and EGF from culture media in three groups after IVM, enzyme-linked immunosorbent assay (ELISA) were performed by using the supernatant obtained from each group (Figure 3). The level of VEGF was significantly higher in the ASC-CM group (70.2 ± 1.1 pg/mL, *p* < 0.05) compared with the control and ASC group (control: 9.0 ± 0.2 pg/mL, ASC group: 59.4 ± 0.3 pg/mL, *p* < 0.05; Figure 3a). Additionally, the bFGF level was significantly higher in the ASC and ASC-CM groups (3.2 ± 0.1 and 3.3 ± 0.1 pg/mL, respectively, *p* < 0.05) compared to the control (2.2 ± 0.1 pg/mL; Figure 3b). As shown in Figure 3c, both ASC and ASC-CM groups showed significantly increased level of IGF-1 (0.6 ± 0.0 and 0.6 ± 0.0 pg/mL, respectively, *p* < 0.05) compared to the control (0.0 ± 0.0 pg/mL). In addition, the IL-10 and EGF concentration were significantly increased in both ASC and ASC-CM groups compared to the control (IL-10: 2.1 ± 0.1 pg/mL, EGF: 42.6 ± 0.4 pg/mL), and ASC-CM group showed a significant increased level of IL-10 and EGF (36.2 ± 0.4 and 50.6 ± 0.9 pg/mL, respectively) compared to the ASC group (25.6 ± 0.3 and 47.8 ± 0.7 pg/mL, respectively; Figure 3d,e).

### 2.3. Quantification of ROS Concentration Derived from Culture Media

**Figure 4:**
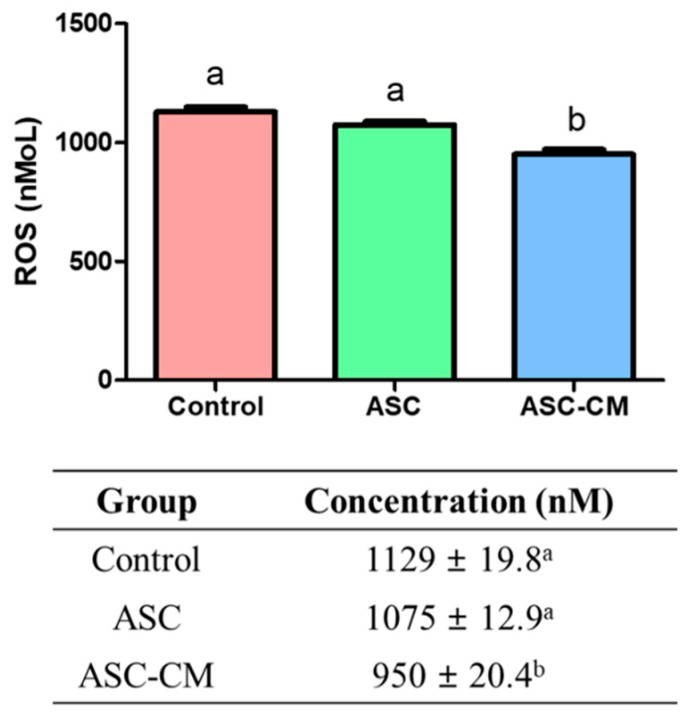
Measurement of reactive oxygen species (ROS) level from culture medium supernatant in three groups. Control; the medium without ASC coculture or ASC-CM. ASC; the medium cocultured with ASC, ASC-CM; the medium cultured with ASC-CM. At least six replications were performed. ^a,b^ Within a column, values with different superscripts are significantly different (*p* < 0.05).

The media from each group were collected to measure the ROS concentrations after IVM by using an Oxiselect^TM^ in vitro ROS/RNS Assay kit (Figure 4). The ROS concentration was measured by the fluorescence intensity at 480 nm excitation/530 nm emission. The production of ROS was significantly decreased in the culture medium derived from the ASC-CM group (950.0 ± 20.4 nM, *p* < 0.05) compared to those of the control and ASC group (control: 1129.0 ± 19.8 nM, ASC group: 1075.0 ± 12.9 nM, *p* < 0.05).

### 2.4. Effects of ASC and ASC-CM during IVM on In Vitro Development of Parthenotes

**Figure 5:**
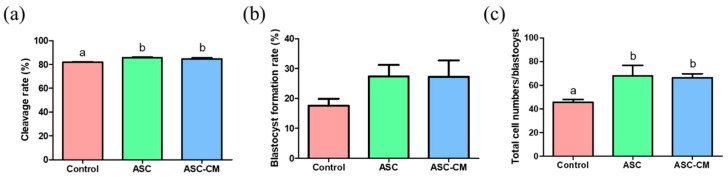
Effect of ASC/ASC-CM on subsequent embryo development after oocyte parthenogenetic activation. (**a**) Cleavage rate of embryos derived from each group, (**b**) blastocyst formation rate of embryos derived from each group, and (**c**) total cell numbers in blastocysts derived from each group. Data are shown as means ± SEM. A total of 445 embryos were used in the experiment. The blastocyst rate was calculated based on total embryos in culture. ^a,b^ Within a column, values with different superscript letters are significantly different (*p* < 0.05).

**Figure 6:**
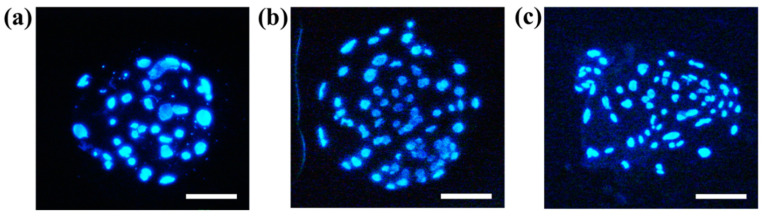
Hoechst 33342-stained embryos at the blastocyst stage using in vitro matured oocytes. (**a**) Blastocyst derived from the control group, (**b**) blastocyst derived from the ASC group, and (**c**) blastocyst derived from the ASC-CM group. The bar represents 100 μm.

The cleavage rate of embryo was evaluated on day 2 of embryos with parthenogenetic activation, and the blastocyst formation rate was assessed on day 7 of the embryos. The ASC and ASC-CM groups showed significantly higher cleavage rates (85.7% ± 0.5% and 84.74% ± 0.7%, respectively, *p* < 0.05) compared with the control group (81.9% ± 0.6%; Figure 5a), whereas there were no significant differences in blastocyst formation rate among three groups (Figure 5b). However, there was an increasing tendency on the blastocyst formation rate in both ASC and ASC-CM groups (27.4% ± 3.8% and 27.3% ± 5.4%, respectively) compared to the control (17.5 ± 2.3%). With respect to the total cell numbers in blastocysts, the ASC and ASC-CM groups showed a significant increase (68.0 ± 8.7 and 66.4 ± 3.3, respectively, *p* < 0.05) compared to the control (45.6 ± 2.3; Figure 5c and Figure 6).

### 2.5. Effects of ASC and ASC-CM on the Relative Gene Expression in Cumulus Cells

**Figure 7:**
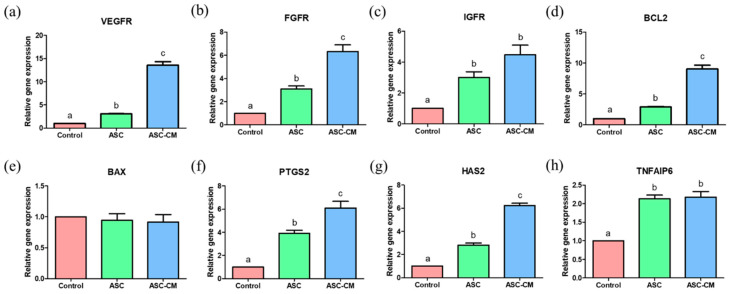
Relative expression of growth factor receptor-related genes (*VEGFR*, *FGFR*, and *IGFR*) (**a**–**c**), apoptosis-related genes (*BCL2* and *BAX*) (**d** and **e**), and cumulus expansion related genes (*PTGS2*, *HAS2*, and *TNFAIP6*) (**f**–**h**) in cumulus cells. Control: cumulus cells cultured without ASC or ASC-CM. ASC; cumulus cells cocultured with ASC during IVM. ASC-CM: cumulus cells cultured with ASC-derived conditioned medium. Data are shown as means ± SEM. ^a,b,c^ Within a column, values with different superscript letters are significantly different (*p* < 0.05). At least three biological replications were performed. A total of 60 randomly selected cumulus–oocyte complexes derived from each group were used for a biological replication. VEGFR; vascular endothelial growth factor receptor, FGFR; fibroblast growth factor receptor, IGFR; insulin growth factor receptor, PTGS2; prostaglandin-endoperoxide synthase 2, HAS2; hyaluronan synthase 2, TNFAIP6; tumor necrosis factor α-induced protein 6.

The real-time PCR was performed to evaluate the relative mRNA transcripts levels associated with growth factor receptors (VEGFR, FGFR, and IGFR), apoptosis (BCL2 and BAX), and cumulus expansion-related genes (PTGS2, HAS2, and TNFAIP6) in cumulus cells derived from COCs after IVM. The mRNA transcripts levels of VEGFR, FGFR, and IGFR were significantly upregulated in the cumulus cells derived from the ASC and ASC-CM groups compared to the control (Figure 7a–c). In particular, the cumulus cells in the ASC-CM group showed significantly higher levels of VEGFR and FGFR compared to those of the ASC group (Figure 7a,b). As for apoptosis gene expression, a significantly increased expression of BCL2 was observed in cumulus cells derived from the ASC-CM group (Figure 7d), whereas there was no significant difference in the BAX level among the three groups (Figure 7e). The PTGS2, TNFAIP6, and HAS2 expression levels were significantly upregulated in cumulus cells from the ASC and ASC-CM groups (Figure 7f–h). Particularly, the expression level of PTGS2 and HAS2 were markedly increased in cumulus cells in the ASC-CM group compared to those in the ASC group (Figure 7f,g).

### 2.6. Effects of ASC and ASC-CM on the Relative Gene Expression in Oocytes

**Figure 8:**
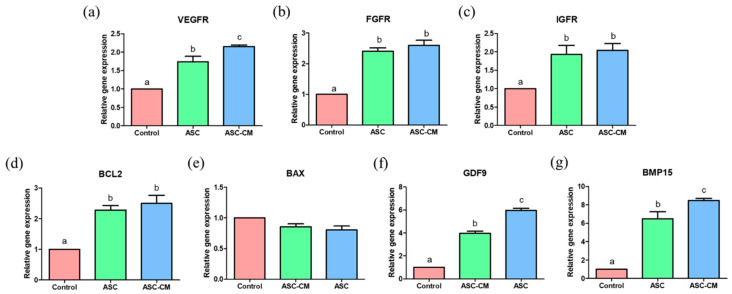
Relative expression of growth factor receptor-related genes (*VEGFR*, *FGFR*, and *IGFR*) (**a**–**c**), apoptosis-related genes (*BCL2* and *BAX*) (**d** and **e**), and oocyte maturation related genes (*GDF9* and *BMP15*) (**f** and **g**) in oocytes. Control: oocytes cultured without ASC or ASC-CM. ASC; oocytes cocultured with ASC during IVM. ASC-CM: oocytes cultured with ASC-derived conditioned medium. Data are shown as means ± SEM. ^a,b,c^ Within a column, values with different superscript letters are significantly different (*p* < 0.05). At least three biological replications were performed. A total of 60 randomly selected cumulus–oocyte complexes derived from each group were used for a biological replication. VEGFR; vascular endothelial growth factor receptor, FGFR; fibroblast growth factor receptor, IGFR; insulin growth factor receptor, GDF9; growth differentiation factor 9, BMP15; bone morphogenetic protein 15.

The real-time PCR was performed to evaluate the relative mRNA transcripts levels relevant to growth factor receptors (VEGFR, FGFR, and IGFR), apoptosis (BCL2 and BAX), and oocyte maturation-related genes (GDF9 and BMP15) in oocytes (Figure 8). The mRNA transcripts levels of VEGFR, FGFR, and IGFR were significantly increased in oocytes from ASC and ASC-CM groups (Figure 8a–c). Especially, the expression level of VEGFR was significantly higher in the ASC-CM group compared to the ASC group (Figure 8a). In regard to apoptosis gene expression, the cumulus cells in ASC and ASC-CM groups showed significantly upregulated levels of BCL2 compared to the control (Figure 8d), however, no significant difference in expression of BAX was observed among the three groups (Figure 8e). Additionally, the GDF9 and BMP15 expression levels were markedly increased in oocytes from ASC and ASC-CM groups. In particular, ASC-CM derived oocytes showed significantly higher levels of GDF9 and BMP15 compared to the ASC group (Figure 8f,g).

### 2.7. Effects of ASC and ASC-CM on the Fluorescence Intensity of GDF9 and BMP15 in Oocytes

**Figure 9:**
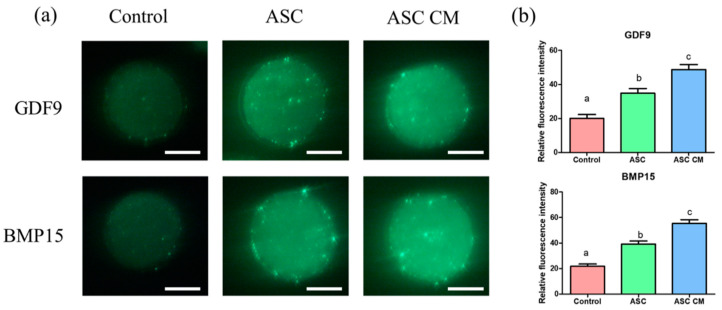
The effect of ASC/ASC-CM on the protein expression of GDF9 and BMP15 in oocytes shown by immunocytochemistry. (**a**) Immunocytochemical analysis of GDF9 (green) and BMP15 (green) in oocytes derived from three groups; control, ASC, and ASC-CM groups. (**b**) The fluorescence intensity for GDF9 and BMP15 in oocytes derived from three groups. Control: oocytes cultured without ASC or ASC-CM. ASC; oocytes cocultured with ASC during IVM. ASC-CM: oocytes cultured with ASC-derived conditioned medium during IVM. Data are shown as means ± SEM. ^a,b,c^ Within a column, values with different superscript letters are significantly different (*p* < 0.05). At least six replications were performed. A total of 3 randomly selected oocytes derived from each group were used for a biological replication. GDF9; growth differentiation factor 9, BMP15; bone morphogenetic protein 15. The bar represents 50 μm.

The immunofluorescence staining was performed to evaluate the protein abundance of GDF9 and BMP15 in each group of oocytes (Figure 9). The protein levels of GDF9 and BMP15 were significantly increased in oocytes derived from ASC and ASC-CM groups compared with the control (*p* < 0.05). Between the two groups (ASC and ASC-CM), the ASC-CM group showed significantly upregulated levels of GDF9 and BMP15 in the oocyte (*p* < 0.05).

## 3. Discussion

The present study suggests that the coculture ASC with porcine oocytes or the supplementation of ASC-CM to the culture medium during IVM markedly improved the oocyte development and increased mRNA transcripts levels related to oocyte maturation, apoptosis, and cumulus expansion. Additionally, it was found that various growth factors/cytokine such as bFGF, VEGF, IGF-1, IL-10, and EGF existed in ASC-CM, which could enhance embryo development competence including cleavage rate and total cell number of the blastocyst. Up to date, the potential effect of human ASC-CM on oocyte development has not been fully investigated. In this regard, the present study suggests that human ASC-CM as enriched supplementary bioactive materials can be applied to ARTs to overcome the present shortcoming of the IVM system.

Culture conditions such as media composition and presence of growth factors are critical to oocyte development during IVM, and subsequent embryo development competence [34,35]. In recent years, many studies have been attempted to establish the optimal condition of IVM by supplementation of growth factors [36,37], extracellular vesicles [38], follicular fluid [5], and mimicking the in vivo microenvironment using a coculture system with fresh oocyte [39], denuded oocyte [40], and somatic cells such as cumulus cells [41] and oviduct cells [42,43]. As the coculture system provides COCs with a similar in vivo microenvironment as closely as possible by transporting multiple paracrine factors into the culture medium [13], the secreted factors derived from the conditioned medium can positively effect on in vitro oocyte development, which is validated by our findings.

According to many previously reported data, stem cells secrete a variety of cytokines, growth factors, proteins, and chemokines that regulate the physiological conditions of cells [44,45]. In particular, a recent study suggested that human mesenchymal stem cells contain various levels of bioactive factors, including EGF, leukemia inhibitory factor (LIF), FGF, and IGF-1, which improve mice oocyte meiotic resumption and the following embryo development [26]. Additionally, numerous paracrine factors such as VEGF, FGF2, IGF-1, and IL-6 exist in human ASC-CM [16,46], and they can substantially improve preimplantation development and efficacy of in vitro fertilization and reduce long-term developmental abnormalities [47]. In line with their results, the presence of the factors in ASC-CM in this study could exert their stimulatory and supportive effects on in vitro oocyte development.

The angiogenesis has been widely associated with physiological processes in the reproductive function such as early luteinization in ovarian follicles [48,49]. The intense expression of *VEGF* mRNA levels was observed in developing follicles during the follicular phase in mice [50] and primates [51]. Additionally, it is noteworthy that primate granulosa cells produce a high concentration level of VEGF than non-luteinized granulosa cells [52], which indicates that VEGF is closely involved in the activation of the folliculogenesis. In line with their results, it is tempting to speculate that the enriched VEGF existed in ASC-CM significantly improved the oocyte quality and subsequent embryo development. In addition, FGF has been considered as a potential paracrine factor that mediates oocyte development. This factor plays a key role in various developmental processes such as regulating cell proliferation, migration, and differentiation in the embryonic stage by a paracrine and/or autocrine manner [53,54]. Additionally, FGF exerts a stimulatory effect in signaling to granulosa/theca cells [55] and promotes glycolysis in cumulus cells with a combination of BMP15 [56]. A recent study demonstrated that bFGF markedly improves cumulus cell expansion with high transcript abundance of an expansion-related gene, and oocyte meiotic resumption in a pig [37]. Additionally, it was indicated that bFGF and its receptor (FGFR) exist in oocytes and follicular cells of all follicular classes, and they have a role in early folliculogenesis in human ovaries [57]. In addition, another study suggested bFGF and FGFR are involved in the growth of the dominant follicle by stimulation of angiogenesis in cows [58]. In this study, there is a significantly increased concentration of bFGF in ASC-CM, and cumulus cells/oocyte showed the high gene expression level of *FGFR* from the ASC-CM group. Therefore, this might indicate that the bFGF derived from ASC-CM activates *FGFR* on COCs, and subsequently regulate the oocyte development during IVM.

It has already been reported that IGF-1 plays an important role in the regulation of ovarian function. A recent study suggested both IGF-1 and IGFR are localized in all ovarian structures including the corpus luteum [59], and the IGF activity in the ovarian follicle influences follicle development, steroidogenesis and oocyte quality [60]. The supplementation of IGF-1 to the IVM culture medium improved the IVM and in vitro fertilization rate in porcine oocytes [61] and this factor stimulates protein kinase A-dependent intracellular mechanisms that involved both meiosis and completion of oocyte nuclear maturation [62]. In the human follicle, *IGFR* mRNA and protein are expressed in preantral follicles at all stages of development, and IGF-1 induces the initiation of follicle growth [63]. In particular, the IGFR is a member of the tyrosine kinase receptor family and it activates the mitogen-activated protein kinase 1/3 pathway [64], which indicates the IGF/IGFR are closely associated with follicular differentiation. In line with their results, it can be suggested that the higher level of IGF-1 derived from ASC-CM could enhance the *IGFR* expression in both cumulus cells and oocytes during IVM, and this could activate the oocyte development.

It has been proved that IL-10 mediates intraovarian cell communication between oocyte and granulosa cells [65], and there is a strong positive correlation between IL-10 and VEGF in the intrafollicular environment during oocyte maturation [66]. Thus, the highest levels of IL-10 together with VEGF in the ASC-CM group might influence oocyte developmental competence in this study. In addition, early evidence substantiates that EGF can enhance oocyte maturation factors such as MAPK, p34cdc2, and cyclin B [67], and promote the growth of oocytes from medium-sized antral follicles [68]. Likewise, the higher level of EGF in ASC-CM compared with other groups might also give a beneficial effect on oocyte development. Overall, numerous kinds of ASC-CM-derived factors such as bFGF, VEGF, IGF-1, IL-10, and EGF contributed to promoting the COCs development and subsequent embryo development by upregulating mRNA transcripts levels.

As ROS are generated during metabolic processes, much attention has been paid to the function of ROS in the oocyte during IVM. Several studies indicate that ROS affects the fertilization rate of the oocyte and embryo developmental competence [69,70]. As in vitro culture conditions contain higher ROS concentration compared with the in vivo environment, and in such conditions, there is a higher ROS level in the culture medium [71,72]. The previous study suggested that the balance between the generation of ROS and the scavenging ability of antioxidants is an essential factor for fertilization in vitro [73]. For instance, ROS production occurs in ovarian follicles during the time of oocyte aspiration, which critically affects a physiological alteration in oocytes [74]. In the present study, the generation of ROS was significantly reduced in the ASC-CM group, which can be implied that the ASC-CM has a potential ROS scavenging activity that could promote the oocyte maturation and subsequent embryo development.

With respect to the potential activity of ASC-CM on oocytes, consequently, ASC-CM significantly promotes the mRNA/protein expression of *GDF9* and *BMP15* in oocytes. As essential members of oocyte–paracrine factors, the GDF9 and BMP15 proteins levels are closely related to their mRNA transcript levels in porcine COCs during IVM [75,76]. These factors act synergistically in COCs development by enhancing the physiological conditions of cumulus cells [77]. For instance, GDF9 and BMP15 mediate the reciprocal communication between oocyte and granulosa cells, inducing oocyte maturation, and cumulus expansion [78]. In the present study, the matured oocytes cultured with ASC-CM showed markedly increased mRNA and protein levels of GDF9/BMP15 and cumulus expansion-related genes, which is consistent with previous studies that showed high mRNA expression of *GDF9* and *BMP15* in matured oocytes activates cumulus cell expansion during IVM by increasing levels of cumulus expansion related genes such as *HAS2*, *TNFAIP6*, and *PTGS2* [75,79,80]. Taking into consideration of the overall gene expression patterns in cumulus cells and oocytes, it can be assumed that ASC-CM can provide sufficient bioactive materials with COCs for enhancing oocyte competence and embryo development. Collectively, this study could provide a basis for highlighting the potential function of ASC-CM on oocyte development, which would provide a new insight in mammalian reproduction including human.

## 4. Materials and Methods

### 4.1. Chemical

All chemicals were obtained from Sigma-Aldrich Co. LLC. (St. Louis, MO, USA) unless otherwise stated.

### 4.2. Isolation and Culture of Adipose-Derived Stem Cells (ASCs)

The procedure for the preparation of human ASC was performed under good manufacturing practices conditions at the R Bio Stem Cell Research Center (Seoul, Korea) with approval from the Life Ethics Committee of the Biostar Stem Cell Institute (RBIO 2015-12-001; Seoul, Korea). The details of specific standards are found in the Code of Federal Regulations, Title 21 (21CFR), and Section 610. The ASC were isolated from disposed human adipose tissues obtained from the lower abdomen of patients, with their agreement, and were cultured as previously described [28]. Briefly, human subcutaneous adipose tissues were collected by simple liposuction from abdominal subcutaneous fat with informed consent and digested with collagenase I (1 mg/mL) under gentle agitation for 60 min at 37 °C. The tissues were then filtered by using a 100-µm nylon sieve and centrifuged at 470× *g* for 5 min. After centrifugation, the tissues were resuspended in a Dulbecco’s modified Eagle’s medium (DMEM; Invitrogen, Grand Island, NY, USA)-based medium containing 0.2 mM ascorbic acid and 10% fetal bovine serum (FBS). The resuspended tissues were recentrifuged at 470× *g* for 5 min. After discarding the supernatant, the cell pellet was collected. The cells were cultured in a DMEM-based medium containing 0.2 mM ascorbic acid and 10% FBS at 37 °C under 5% CO_2_. After 12 h, the culture medium was changed to keratinocyte-SFM (Invitrogen)-based medium containing 0.2 mM ascorbic acid, 0.09 mM calcium, 5 ng/mL recombinant epidermal growth factor (rEGF), and 5% FBS. The cells were cultured for 4–5 days until confluent. They were subcultured in keratinocyte-SFM-based medium containing 0.2 mM ascorbic acid, 0.09 mM calcium, 5 ng/mL rEGF, and 5% FBS after reaching 90% confluency. The cells were cryopreserved in 10% dimethyl sulfoxide containing a freezing solution until further experiments.

### 4.3. Preparation of Human Adipose-Derived Stem Cells Conditioned Medium (ASC-CM)

To prepare the ASC-CM, ASC were thawed and cultured until they reached about 70–80% confluency in a 12-well plate with the defined keratinocyte-SFM-based medium at 37 °C under 5% CO_2_ in the air. Then, the culture medium was changed with serum-free Dulbecco’s modified Eagle’s medium (DMEM; Invitrogen, Grand Island, NY, USA). After 24 h of starvation, the supernatant was obtained and centrifuged at 13,000× *g* for 5 min at 4 °C. Then, the media were filtered by using a 0.22 µm filter.

### 4.4. In Vitro Maturation of Oocytes by Coculture with Human Adipose-Derived Stem Cells (ASC) and ASC Conditioned Medium (ASC-CM)

Porcine ovaries were obtained from sows at a local slaughterhouse, and the ovaries were transported to the laboratory in 0.9% normal saline at 32–35 °C within 3 h. The COCs were collected from 3 to 6 mm diameter follicles with an 18-gauge needle on a 10 mL disposable syringe. Then, the COCs were washed three times in the washing medium containing 9.5 g/L of TCM-199, 2 mM sodium bicarbonate, 10 mM HEPES, 0.3% polyvinyl alcohol, 5 mM sodium hydroxide, and 1% penicillin–streptomycin (Invitrogen). Next, the COCs were selected on the basis of the following morphological features: three or more compact multilayers of cumulus cells and homogeneous cytoplasm. The COCs were cultured in IVM medium containing TCM-199 supplemented with 0.57 mM cysteine, 0.91 mM sodium pyruvate, 5 μL/mL insulin–transferrin–selenium solution 100× (Invitrogen), 10 IU/mL equine chorionic gonadotropin (eCG), and 10 IU/mL human chorionic gonadotropin in a culture plate.

To perform the experiment, the COCs were randomly divided into three groups: control, ASC coculture group, and ASC-CM group. For the ASC coculture experiment, ASC were used when they had reached about 70% confluency in a 12-well plate with the medium. The medium was changed with an IVM medium when coculture was performed. The 12-well plates were supported with 1.0 μm Transwell polyester membrane inserts (400 μL media per inserts; Corning Inc., Pittston, PA, USA) to allow mutual communication between porcine oocytes and ASC for a total of 44 h at 39 °C in a humidified atmosphere of 5% CO_2_. The transwell system provides COCs with paracrine factors secreted from ASC through the microporous membranes under the transwell inserts. The distance of intercellular communication was approximately 2 mm.

For the ASC-CM experiment, freshly collected ASC-CM (combine IVM medium and ASC-CM in the ratio of 1 to 1) were used in a culture plate during IVM. The COCs were cultured for 22 h with 10 IU/mL eCG and then washed twice in the eCG-free medium. Subsequently, the COCs were cultured for an additional 22 h in IVM medium without eCG. The COCs were denuded by using 0.1% hyaluronidase with gentle pipetting after 44 h culture for IVM. To evaluate the in vitro maturation rate, the extrusion of the first polar body (Metaphase II) from oocytes was assessed under the stereomicroscope (TE2000-S; Nikon, Tokyo, Japan) with magnification ×80. After collecting denuded oocytes, the medium was centrifuged at 1975× *g* for 2 min to collect the cumulus cells. Then, the pellets were washed in phosphate buffer saline (PBS) and immediately stored at −80 °C until used for further experiments.

### 4.5. Evaluation of Cumulus Expansion Degree

The cumulus expansion degree in oocytes was assessed by the morphology of COCs after 44 h of IVM. In brief, a degree of 0 indicated no expansion, along with the detachment of cumulus cells from the oocyte, suggesting a partially or fully denuded oocyte. A degree of 1 represented no expansion but compacted cumulus cells remained around the oocyte. A degree of 2 represented the expansion of only the outermost layers of cumulus cells. A degree of 3 indicated that all cell layers were expanded except the corona radiata (cells most proximal to the oocyte), and a degree of 4 showed the maximum degree of expansion including the corona radiata.

### 4.6. ELISA Analysis

The concentrations of the basic fibroblast growth factor (bFGF, MyBioSource, San Diego, CA, USA), VEGF (R&D Systems, Minneapolis, MN, USA), insulin growth factor-1 (IGF-1, R&D Systems, Minneapolis, MN, USA), IL-10 (R&D Systems, Minneapolis, MN, USA), and epidermal growth factor (EGF, R&D Systems, Minneapolis, MN, USA) in the supernatant obtained from three groups after IVM were measured by ELISA. The assay was performed according to the manufacturer’s instructions. In brief, standard solutions and samples were added to each well in the designated well on the plates and incubated for 1–2 h at room temperature. After aspirating the solution in each well, the wash buffer was applied to each well for three to five times to remove unbound antigen. Then, the proper amounts of conjugates were added to each well and incubated for 1–2 h at room temperature. After incubation, each well was washed three to five times with a wash buffer. The substrate solutions were added to each well and incubated for 30 min at room temperature with protection from intense light. Lastly, stop solutions were added to each well with gentle tapping and the spectroscopic absorbance of each well was measured with a microplate reader (Tecan Sunrise, Hayward, CA, USA) at 450 nm excitation/590 nm emission.

### 4.7. Assessment of In Vitro ROS Levels in Media

ROS are molecules that contain oxygen and that easily reacts with other molecules in a cell, and they induce deleterious effects to cells. ROS concentration was determined by an OxiselectTM in vitro ROS/RNS Assay kit (Cell Biolabs, San Diego, CA, USA). The assay was performed according to the manufacturer’s instructions. Briefly, the media from each group were collected to measure the free radical presence in the samples. All samples were transferred into 1.5 mL tubes and centrifuged at 10,000× *g* for 5 min. Each sample was added to wells of the designated plate. Then, 50 μL of the catalyst was added to each well followed by incubation for 5 min at room temperature. Lastly, 100 μL of DCFH solution was added to each well, and incubation was performed for 15 min at room temperature. The fluorescence intensity was measured using a fluorescence plate reader at 480 nm excitation/530 nm emission (Sunrise, Tecan, Hayward, CA, USA).

### 4.8. Parthenogenetic Activation and In Vitro Culture of Parthenotes

The COCs of each group were denuded by gently pipetting with 0.1% hyaluronidase after 44 h of IVM, and the oocytes were washed in Tyrode’s albumin lactate pyruvate (TALP) medium. The denuded oocytes were gradually equilibrated in an activation medium containing 0.28 M mannitol, 0.5 mM HEPES, 0.1 mM MgSO_4_, and 0.1 mM CaCl_2_. Then, the oocytes with homogeneous cytoplasm were placed between two electrodes filled with activation solution in a chamber connected with a BTX Electrocell Manipulator ECM 2001 (BTX Inc., San Diego, CA, USA). With a single direct current pulse of 1.5 kV/cm for 60 μsec, the oocytes were activated. Thereafter, electrically activated oocytes were washed in porcine zygote medium-5 (PZM-5; Funakoshi Corporation, Tokyo, Japan), and were transferred into the drops of PZM-5 covered with prewarmed mineral oil in a 4-well dish. The oocytes were cultured for 7 days at 39 °C in a humidified atmosphere of 5% O_2_, 5% CO_2_, and 90% N_2_. The day of parthenogenetic activation (PA) was considered as day 0. The cleavage rate of embryos was assessed on day 2 under a stereomicroscope. On day 7, the blastocyst formation rate was evaluated. The blastocysts were stained with 5 μm/mL of Hoechst 33,342 for 7 min to count the total cell number in blastocysts. After washing in the TALP medium, stained blastocysts were mounted on a glass slide in a drop of glycerol with gentle compression using a cover slip. The total cell was counted using a fluorescence microscope (Nikon Corp., Tokyo, Japan) at ×400 magnification.

### 4.9. Total RNA Extraction and cDNA Synthesis

Total RNA was extracted from cumulus cells and oocytes derived from COCs using the Easy-spinTM (DNA-free) Total RNA Extraction Kit (iNtRON Biotechnology Inc., Kyunggi, Korea) according to the manufacturer’s instructions. The total RNA concentration was determined by using spectrophotometry (NanoDrop 2000, Thermo Fisher Scientific Inc., Waltham, MA, USA) and the samples were reverse transcribed into cDNA using amfiRivert II cDNA Synthesis Premix (GenDEPOT, Barker, TX, USA) following the manufacturer’s instructions.

### 4.10. Real-Time PCR

The primers for VEGFR, FGFR, IGFR, BCL2, BAX, GDF9, BMP15, PTGS2, HAS2, TNFAIP6, and GAPDH genes were designed from sequences of porcine genes obtained from NCBI; all primer sequences were standardized using a standard curve and are listed in Table 1. The expression of each target gene was quantified relative to that of the internal control gene (GAPDH). Real-time PCR was performed using an ABI 7300 Real-Time PCR System (Applied Biosystems, Foster City, CA, USA) according to the manufacturer’s instructions with minor modification. Briefly, the total volume PCR reaction mixture was 20 μL in a real-time PCR plate (MicroAmp optical 96-well reaction plate, Singapore). The mixture was composed of 2 μL cDNA, 0.4 μL forward primer, 0.4 μL reverse primer, 10 μL SYBR Green interaction dye (Takara Bio USA Inc., Mountain View, CA, USA), and 7.2 μL nuclease-free water. The reactions were carried out for 40 cycles with the following parameters of cycles: (1) denaturation (95 °C for 30 s), (2) annealing (55 °C for 30 s), and (3) extension (72 °C for 30 s).

### 4.11. Immunocytochemistry

Immunofluorescence staining was performed to evaluate the effect of ASC and ASC-CM on the expression of GDF9 and BMP15 in oocytes. In brief, oocytes were washed three times in PBS containing 0.2% PVA. Then, they were fixed with 4% paraformaldehyde (*w*/*v*) in PBS for 30 min at room temperature. After incubation, the fixed oocytes were washed three times in PBS, and oocytes were permeated with 1% (*v*/*v*) Triton X-100 in PBS for 2 h at room temperature. The samples were washed and blocked with 2% bovine serum albumin in PBS for 4 h at 4 °C. The primary antibodies for GDF9 (1:200; ab93892, Abcam, MA, USA) and BMP15 (1:200; PA5–34401, Thermo Fisher Scientific, IL, USA) were treated to the oocytes, and incubation was performed at 37 °C overnight. After washing three times in PBS with 2% BSA, they were incubated with a secondary antirabbit polyclonal antibody (1:200; ab6717, Abcam) for 3 h at room temperature. The samples were washed several times with PBS and they were mounted on glass slides. Images were captured under a fluorescence microscope (Nikon Corp.) with the same exposure times and adjustments at magnification ×100. The fluorescence intensities of GDF9 and BMP15 were measured by Image J software (version 1.46r; National Institutes of Health, USA).

### 4.12. Statistical Analysis

All data were analyzed by a one-way ANOVA followed by Tukey’s multiple comparison test using GraphPad Prism 5.0 (Graphpad, San Diego, CA, USA). Values are shown as means ± standard error of the mean. *p* values < 0.05 were considered to be statistically significant. All experiments were performed with a minimum of three independent replicates.

## 5. Conclusions

To the best of the author’s knowledge, this study first demonstrated the efficiency of ASC-CM on porcine oocyte development during IVM. Moreover, multiple growth factors/cytokine that existed in ASC-CM were actively involved in the COCs development by regulating mRNA/protein expression. Taken together, this finding provides a strong basis for the further establishment of optimal IVM conditions and highlights the potential function of ASC-CM as a paradigm to establish a reliable system for ARTs.

## Figures and Tables

**Table 1:** Sequence-specific primers used for quantification of differentially expressed transcripts.

Gene	Primer Sequences (5′ → 3′)	GenBank No.	Product Size (bp)
*GAPDH*	F-CTTCCACTTTTGATGCTGGGG R-TCCAGGGGCTCTTACTCCTT	NM_001206359.1	145
*VEGFR*	F-AAGCCATTCCGATAACAACG R-GCTGTTTTCGATGCTTCACA	NM_021065525.1	173
*FGFR*	F: TCATCTGCCTGGTTGTGGTC R: CGCAGCCACGTAAACTTCTG	NM_001099924.2	140
*IGFR*	F: CCCAATGGCAACCTGAGCTA R: TCCTCGACATCAATGGTGCC	NM_214172.1	137
*BCL2*	F-AGGGCATTCAGTGACCTGAC R-CGATCCGACTCACCAATACC	NM_214285	193
*BAX*	F-TGCCTCAGGATGCATCTACC R-AAGTAGAAAAGCGCGACCAC	XM_003127290	199
*GDF9*	F-ACATGACTCTTCTGGCAGCC R-ACCCTCAGACAGCCCTCTTT	NM_001001909.1	140
*BMP15*	F-AGCTCTGGAATCACAAGGGG R-ACAAGAAGGCAGTGTCCAGG	NM_001005155.1	123
*PTGS2*	F-TGGGGAGACCATGGTAGAAG R-CTGAATCGAGGCAGTGTTGA	NM_214321.1	142
*HAS2*	F-AGTTTATGGGCAGCCAATGTAGTT R-GCACTTGGACCGAGCTGTGT	AB050389	101
*TNFAIP6*	F-AGAAGCGAAAGATGGGATGCT R-CATTTGGGAAGCCTGGAGATT	NM_001159607	106

## Data Availability

The data presented in this study are available on request from the corresponding author. The data are not publicly available due to privacy/ethical restrictions.

## Abbreviations

ARTs	Assisted reproductive techniques
FBS	Fetal bovine serum
PBS	Phosphate buffer saline
TALP	Tyrode’s albumin lactate pyruvate
TNF	Tumor necrosis factor
CM bFGF	Conditioned medium Basic fibroblast growth factor
VEGF	Vascular endothelial growth factor
IGF-1	Insulin growth factor 1
IL-10	Interleukin 10
EGF	Epidermal growth factor
IVM	In vitro maturation
IVC	In vitro culture
ROS	Reactive oxygen species
FGFR	Fibroblast growth factor receptor
IGFR	Insulin growth factor 1 receptor
PTGS2	Prostaglandin-endoperoxide synthase 2
TNFAIP6	Tumor necrosis factor α-induced protein 6
HAS2	Hyaluronan synthase 2
GDF9	Growth differentiation factor 9
BMP15	Bone morphogenetic protein 15
ELISA	Enzyme-linked immunosorbent assay
COCs	Cumulus-oocyte complexes
PA	Parthenogenetic activation
DMEM	Dulbecco’s Modified Eagle’s Medium
TCM-199	Tissue culture medium 199
eCG	Equine chorionic gonadotropin
hCG	Human chorionic gonadotropin
PZM-5	Porcine zygote medium-5
